# The Immunogenic and Immunoprotective Activities of Recombinant Chimeric *T. gondii* Proteins Containing AMA1 Antigen Fragments

**DOI:** 10.3390/vaccines8040724

**Published:** 2020-12-02

**Authors:** Justyna Gatkowska, Katarzyna Dzitko, Bartłomiej Tomasz Ferra, Lucyna Holec-Gąsior, Malwina Kawka, Bożena Dziadek

**Affiliations:** 1 Department of Molecular Microbiology, Faculty of Biology and Environmental Protection, University of Lodz, 90-237 Łódź, Poland; katarzyna.dzitko@biol.uni.lodz.pl (K.D.); malwina.kawka@biol.uni.lodz.pl (M.K.); bozena.dziadek@biol.uni.lodz.pl (B.D.); 2 Department of Molecular Biotechnology and Microbiology, Faculty of Chemistry, Gdańsk University of Technology, 80-233 Gdańsk, Poland; barferra@pg.edu.pl (B.T.F.); lucholec@pg.edu.pl (L.H.-G.)

**Keywords:** chimeric antigen, immunogenicity, immunoprotection, *Toxoplasma gondii*, murine experimental model

## Abstract

Toxoplasmosis, one of the most common parasitoses worldwide, is potentially dangerous for individuals with a weakened immune system, but specific immunoprophylaxis intended for humans is still lacking. Thus, efforts have been made to create an efficient universal vaccine for both animals and humans to overcome the shortcomings of currently used treatment methods and protect all hosts against toxoplasmosis. The current work represents a relatively new approach to vaccine development based on recombinant chimeric *Toxoplasma gondii* antigens. In the present research, three tetravalent chimeric proteins containing different portions of the parasite’s AMA1 antigen—AMA1^domain^ ^I^-SAG2-GRA1-ROP1_L_ (A^N^SGR), AMA1^domains^ ^II,^ ^III^-SAG2-GRA1-ROP1_L_ (A^C^SGR) and AMA1^full^ ^protein^-SAG2-GRA1-ROP1_L_ (A^F^SGR)—were tested for their immunogenic and immunoprotective capacities. All tested proteins were immunogenic, as evidenced by the triggering of specific humoral and cellular immune responses in vaccinated C3H/HeOuJ mice, defined by the production of specific IgG (IgG1/IgG2a) antibodies in vivo and synthesis of key Th1/Th2 cytokines by *Toxoplasma* lysate antigen-stimulated splenocytes in vitro. Although all tested preparations provided partial protection against chronic toxoplasmosis in immunized and *T. gondii*-challenged mice, the intensity of the generated immunoprotection depended on the fragment of the AMA1 antigen incorporated into the chimeric antigen’s structure.

## 1. Introduction

Apicomplexan parasites are responsible for many parasitoses, including some of great medical concern or high veterinary importance. Among these parasites, cosmopolitan protozoa can be found, such as *Toxoplasma gondii*, *Criptosporidium* spp., *Isospora* spp., *Neospora* spp., *Eimeria* spp., and *Babesia* spp., but also others, such as *Plasmodium* spp. and *Theileria* spp. [1,2,3,4,5,6]. There is no licensed vaccine against any human parasitic disease, including *T. gondii* invasion [7,8], which, under certain circumstances, may pose a serious threat to the health and even the life of infected hosts, and thus presents a great challenge for research centers looking for new and effective drugs and/or vaccines to prevent the possible adverse effects of toxoplasmosis [9,10,11,12,13].

People typically become infected by three principal routes of transmission: foodborne, animal-to-human (zoonotic), and mother-to-child (congenital) [14,15,16], and there are rare instances of infection following receipt of an organ from a *Toxoplasma*-positive donor or infected blood administered via transfusion [17]. The main route of human and carnivore animal infection by *Toxoplasma gondii* is the consumption of raw or undercooked meat containing the tissue form of the parasite (cysts) with enclosed slowly dividing bradyzoites [18]. Infection can also occur through consumption of food contaminated with the feces of *Felidae*, in which excreted oocysts with *T. gondii* sporozoites may be found. Environmentally stable oocysts can be a source of infection in all warm-blooded animal species (both herbivores and carnivores), including humans. Consumption of either environmental oocysts or tissue cysts results in tachyzoites release in the small intestine of the host, which are responsible for the acute phase of disease and ultimately colonization of the infected organism by bradyzoites during the chronic stage without the possibility of parasite elimination from the infected host [19,20]. *Toxoplasma* is an obligate intracellular parasite that, after infection, forms parasitophorous vacuole (PV) inside the host cell that is responsible for mediating the communication process between the parasite and the host cell and enables tachyzoites to multiply by endodyogeny [21]. In addition, this specialized structure protects daughter tachyzoite cells from the components of host immunity, which involves innate and adaptive immune responses. In immunocompetent individuals, the acute phase of disease passes into a chronic phase characterized by the formation of tissue cysts [11,22].

In cases of impaired immunity, bradyzoites can again be released from cysts and, without the pressure of a host immune response, spread throughout the organism or be responsible for local necrotic phenomena. A particularly dangerous and life-threatening infection in newborns is congenital toxoplasmosis, which occurs mainly after primary infection of a pregnant woman. However, cases of tachyzoite transmission via the placenta in women infected shortly before pregnancy, immunocompromised women undergoing reactivation and women chronically infected with one *T. gondii* serotype and developing acute-phase disease after invasion by a second *Toxoplasma* serotype are well described in the medical literature. Infants with severe congenital toxoplasmosis usually show symptoms at birth or develop symptoms during the first six months of life [23,24]. The observed symptoms of congenital toxoplasmosis include premature birth, abnormally low birth weight, eye damage, anemia, feeding difficulties, swollen lymph nodes, macrocephaly, microcephaly, vision problems, hearing loss, motor and developmental delays, hydrocephalus, intracranial calcification, evidence of areas with brain damage caused by parasite multiplication and mild to severe mental retardation [25,26,27].

Drugs designated for other disease treatments, which possess antibacterial (sulfadiazine, spiramycin and clindamycin) or antimalarial activity (pyrimethamine and atovaquone), are currently used to treat toxoplasmosis. First-line therapy is based on combination of pyrimethamine and sulfadiazine administered with folinic acid to reduce the bone marrow suppression caused by both antifolates. In case of sensitivity, sulfadiazine may be replaced by clindamycin, however the treatment has similar toxicity. Another therapeutic options are trimethoprim-sulfamethoxazole and atovaquone or azithromycin combination with pyrimethamine or sulfadiazine, used for both the treatment and prophylaxis of toxoplasmosis in cases when first-line therapy is contraindicated [9,10].

Noteworthy, proposed therapeutic strategies usually cause toxic side effects, require prolonged treatment for weeks to over a year, do not protect against recurrence of toxoplasmosis due to a lack of efficacy against bradyzoites enclosed in tissue cysts and do not achieve satisfactory therapeutic concentrations in the brain and eye tissues [28,29]. Therefore, efforts have been made to develop new drugs specifically for toxoplasmosis treatment [30,31,32,33]. Another approach involves the construction of an efficient universal vaccine to protect not only humans but also food-producing livestock from *T. gondii* invasion [8,11,12,13]. Protective veterinary vaccines have a significant impact not only on animal health and production but also on human health by increasing the safe food supply and preventing animal-to-human transmission [34].

Murine experimental toxoplasmosis is a well-characterized and commonly used model for both drug and vaccine development. Therefore, efforts to create an effective vaccine using an experimental mouse model are a trend in the development of vaccines, such as recombinant subunit vaccines containing one or multiple target antigens. Chimeric proteins are used in this approach and constitute a fairly new and promising tool for the construction of products eliciting specific immunoprophylaxis. Of note, recombinant proteins represent one of the most common strategies in vaccine development and have already been successfully employed clinically in several commercially available vaccines against different infectious agents.

Thus, the aim of the study was to evaluate the immunogenic and protective efficacies of three recombinant chimeric *T. gondii* proteins containing different fragments of the AMA1 antigen. The experimental design of the present work was based on our previous observations, which led to the selection of one particularly promising chimeric antigen constituting a potential object for further research on specific anti-*Toxoplasma* prophylaxis due to its high immunogenic and immunoprotective potential leading to high but not complete protection against cyst formation in mice [35]. Thus, an attempt was made to further boost the immunoprotective capacity of the SAG2-GRA1-ROP1_L_ (SGR) fusion protein by incorporating different portions of the *T. gondii* apical membrane antigen−1 (AMA1) into its structure. AMA1 of the parasite, as the microneme protein, is engaged in the initial stages of parasite invasion including recognition of the target cell, attachment to the target cell and subsequent entry into the target cell. The invasion process executed by the parasite relies on several critical stages, and AMA1 is involved in the formation of the moving junction (MJ) complex, which is one of the most crucial structures during parasite entry into a cell [36,37,38]. Moreover, the antigen has been shown to exhibit immunogenic and protective capabilities against acute and chronic toxoplasmosis in laboratory mice [39,40,41]. Taking into account the diverse roles of AMA1 fragments including the N-terminal portion corresponding to domain I and the C-terminal fragment corresponding to domains II and III and the full-length antigen, these constructs were added to the SGR core to form the following tetravalent preparations: AMA1^domain I^-SAG2-GRA1-ROP1_L_ (A^N^SGR), AMA1^domains II,III^-SAG2-GRA1-ROP1_L_-(A^C^SGR) and AMA1^full protein^-SAG2-GRA1-ROP1_L_-(A^F^SGR). These recombinant proteins were then thoroughly tested as prospective vaccine candidates using an experimental mouse model.

## 2. Materials and Methods

### 2.1. Mice

In vivo experiments were performed with male C3H/HeOuJ mice (8–12 weeks of age) from Charles River Laboratories (Wilmington, MA, USA) that were bred in the animal facility of the Faculty of Biology and Environmental Protection, University of Lodz under specific pathogen-free (SPF) conditions. Animals (2–3/cage) were kept in stable conditions: temperature of 21 °C +/−0.5 to +/−5%, 55% humidity, 12/12–h dark/light cycle, 15–20 air exchanges per hour. Mice had free access to water and standardized feed *ad libitum*. All materials, including bedding, food and water were autoclaved before use. All mice were acclimated for 3–5 days before the start of experimental procedures and monitored throughout the experiment.

### 2.2. Ethical Statement

All experimental procedures involving animals were caried out according to guidelines provided and approved by the Polish Local Ethics Commission for Experiments on Animals in Łódź (Agreement 66/ŁB79/2017), which operates on the basis of the legal act on the welfare of animals used for educational and scientific purposes.

### 2.3. Parasites

The parasite strains used in the study, included the low-virulence *T. gondii* DX strain and highly virulent RH strain (50174™, ATCC^®^, Manassas, VA, USA), which were maintained in vivo through laboratory mice and in vitro on human foreskin fibroblasts (Hs27, CRL−1634™, ATCC^®^, Manassas, VA, USA), respectively, as described previously [35]. The DX strain was used to induce experimental *T. gondii* infection in mice, while the RH strain was utilized as a source of native parasite antigens (*Toxoplasma gondii* lysate antigen, TLA) and DNA for cloning procedures involved in recombinant antigen production.

### 2.4. Production of Recombinant Chimeric Proteins

Tetravalent recombinant chimeric proteins containing different fragments of the *T. gondii* AMA1 antigen including AMA1^domain I^-SAG2-GRA1-ROP1_L_ (A^N^SGR), AMA1^domains II,III^-SAG2-GRA1-ROP1_L_ (A^C^SGR) and AMA1^full protein^-SAG2-GRA1-ROP1_L_- (A^F^SGR) were produced, purified and analyzed according to procedures described previously [35,42].

### 2.5. Mouse Immunization and Challenge

Mice were randomly allocated to cages, which were then randomly assigned to experimental groups: immunized only (4–5 mice per group) or immunized and challenged (8–10 mice per group) according to a procedure used and described previously [35]. Each mouse received 10 µg of test antigen with incomplete Freund’s adjuvant (IFA) (Merck KGaA, Darmstadt, Germany), and immune parameter assessment and parasite challenge were carried out two weeks after the last immunization. All animals under study, from both control and test groups, underwent experimental procedures at the same time. All procedures were carried out by the same experimenters and only one of them was aware of animals’ allocation. As previously described, mice injected with PBS/IFA constituted a negative control.

### 2.6. Brain Cyst Enumeration

The assessment of the number of tissue cysts present in the brain of control and immunized *T. gondii*-challenged mice was performed as described previously [35], and the obtained results were calculated to represent the cyst burden in each mouse brain.

### 2.7. Determination of the Specific IgG response and Isotype Profile after Immunization

As described previously [35,43], the humoral response of mice after immunization was defined by the level of IgG antibodies (serum diluted 1:500) recognizing a native *T. gondii* antigen (TLA) and the titers of chimeric antigen-specific IgG1 and IgG2a isotypes (serum diluted from 1:100 to 1:204,800) present in immune and control mouse sera.

### 2.8. In Vitro Splenocyte Proliferation

The proliferative response of splenocytes isolated from mice and stimulated in vitro with TLA (at a final concentration of 10 µg/mL) was assessed as described previously [35,43]. The culture medium and concanavalin A (Merck KGaA, Darmstadt, Germany) served as negative and positive controls for proliferation, respectively.

### 2.9. Determination of Cytokine Production by Stimulated Splenocytes

The concentrations of cytokines in the supernatants of in vitro-stimulated splenocytes from immunized and control mice were determined with commercially available OptEIA™ ELISA sets (BD Biosciences, San Jose, CA, USA) according to the manufacturer’s instructions.

### 2.10. Statistical Analysis

Data obtained from all sacrificed animals were analyzed and obtained results, excluding the cyst burden and IgG isotype titers, are presented as the mean value for each experimental group ± standard deviation. For the IgG isotypes, the amounts of chimeric antigen-specific IgG1 and IgG2a antibodies are expressed as the highest serum dilution of each tested mouse that generated an OD value exceeding 0.3 [35,43]. All statistical analyses were performed with Statistica, ver. 12.0 (TIBCO Software Inc., Palo Alto, CA, USA) using the Mann-Whitney U test, and the results were considered to be statistically significant at *p* < 0.05.

## 3. Results

### 3.1. Protection against Cyst Formation in Mice

**Figure 1:**
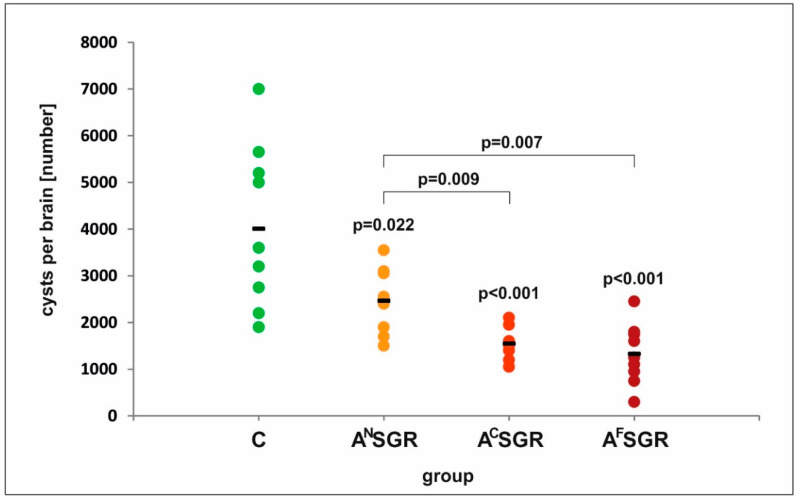
Cyst numbers in control and immunized mice challenged with *T. gondii*. *p* values are provided above the dots to indicate significant differences in the cyst burden between immunized mice (A^N^SGR: *n* = 9; A^C^SGR: *n* = 8; A^F^SGR: *n* = 9) and control mice (C: *n* = 10); statistically significant differences between immunized groups are marked with brackets; and the bars represent the mean cyst numbers in experimental groups.

As indicated in Figure 1, immunization with any of the three chimeric antigens tested, regardless of the portion of the AMA1 antigen incorporated into the chimeric antigen structure, resulted in a statistically significant decrease in the cyst burden determined one month after *T. gondii* challenge. However, the provided protection rate depended strongly on the AMA1 fragment present in the chimeric antigen. The protein containing the N-terminal fragment of AMA1 including domain I of the antigen (A^N^SGR) was the least efficient in preventing tissue cyst formation, as mice immunized with this chimeric antigen exhibited only a 38% reduction in the cyst load compared to nonimmunized controls. The recombinant protein composed of the C-terminal portion of AMA1 including domains II and III of the antigen (A^C^SGR) exhibited a statistically higher immunoprotective capacity (*p* = 0.009) of 61%. The tetravalent chimeric antigen with the full-length AMA1 antigen (A^F^SGR) provided the best protection against chronic toxoplasmosis (67%) compared to no immunization, and this value was significantly higher than the value for the protection provided by the A^N^SGR protein (*p* = 0.007). Although the tetravalent variant containing the full-length AMA1 antigen seemed more potent than the A^C^SGR protein, the difference in the provided protection rate between the two groups was not statistically significant (*p* = 0.469).

### 3.2. Analysis of the IgG production and Isotype Profile

**Figure 2:**
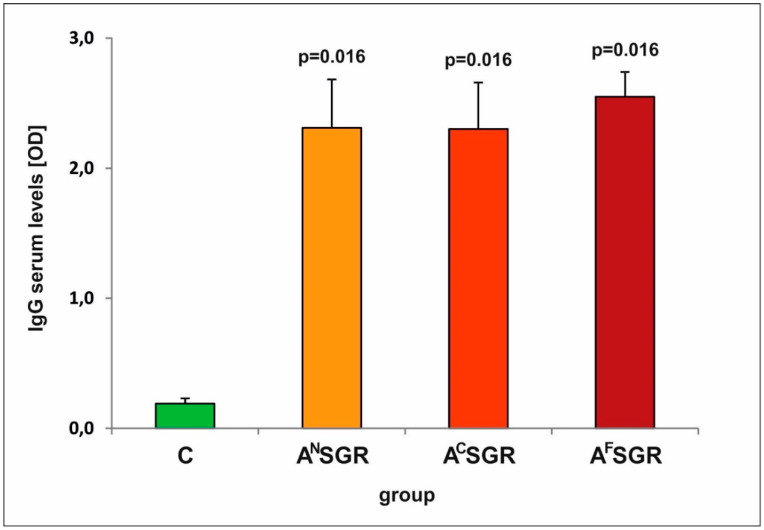
Reactivity of specific IgG antibodies present in sera from control (C: *n* = 5) and immunized (A^N^SGR, A^C^SGR, A^F^SGR: *n* = 4) mice with TLA. *p* values are provided above the bars to indicate significant differences in reactivity between immunized mice and control mice (C).

The evaluation of the specific humoral response in mice after immunization defined by the serum IgG activity specific for a native *T. gondii* antigen (TLA) revealed that all tested tetravalent antigens containing different fragments of the AMA1 protein were capable of inducing IgG antibodies recognizing their native equivalents present in TLA (Figure 2). Interestingly, there were no statistically significant differences in IgG levels among the immunized groups despite slightly higher values being obtained in mice injected with the A^F^SGR antigen (*p* > 0.100, for all tested antigenic variants).

Analysis of IgG isotypes in the serum (Table 1) showed that all immunized mice, regardless of the antigen used, produced recombinant antigen-specific IgG1 and IgG2a antibodies. Comparison of the obtained titer values revealed quite similar IgG1 antibody responses within and among experimental groups (*p* > 0.500). 

In contrast, the production of IgG2a antibodies differed greatly among individuals, even within one group. Notably, despite the observed individual differences among tested animals, the IgG2a amounts in the serum of A^F^SGR-immunized mice were significantly higher than those in the serum of mice in the A^C^SGR-injected group (*p* = 0.029). No statistically significant differences in IgG2a synthesis were noticed in the comparisons among the mice immunized with the tetravalent chimeric antigen containing the N- and C-terminal regions of the AMA1 antigen and the A^N^SGR- and A^F^SGR-injected animals (*p* > 0.400). Generally, the comparison of synthetized IgG isotypes showed a statistically significant predominance of IgG1 antibodies in all experimental groups (*p* = 0.029, for all antigenic variants). All control sera from PBS/adjuvant-injected mice were negative, showing no specific reactivity for any of the recombinant antigens tested at a dilution of 1:100.

### 3.3. Cellular Response Induced by Vaccination

The results obtained in an in vitro splenocyte proliferation assay after TLA stimulation (Table 2) showed that cells from all immunized mice exhibited better proliferation in TLA-treated cultures than in untreated cultures (*p* < 0.001). Comparison of the specific native *T. gondii* antigen-induced splenocyte proliferative response between immunized and control mice revealed that immunization with the tetravalent chimeric antigen containing the full-length AMA1 protein led to a significantly higher proliferative response in cells in vitro (^a^*p* = 0.036). Splenocytes from A^N^SGR-vaccinated mice showed no proliferative response to TLA stimulation compared to TLA-treated control cells, and their proliferative activity in response to TLA stimulation was significantly lower than that of cells from mice in the other immunized groups (*p* = 0.040 for comparison to cells from A^C^SGR-vaccinated mice, *p* = 0.017 for comparison to cells from A^F^SGR-vaccinated mice). In the case of the A^C^SGR-immunized group, the proliferation in response to TLA was higher than for TLA-stimulated control splenocytes; however, the noticed difference oscillated on the border of statistical significance (*p* = 0.067) but was significant when a *t*-test was applied (*p* = 0.044). Notably, there were no statistically significant differences in proliferation after TLA stimulation between A^C^SGR- and A^F^SGR-vaccinated mice (*p* = 0.707).

### 3.4. Determination of IL−2, IFN-γ and IL−10 In Vitro Synthesis

**Figure 3:**
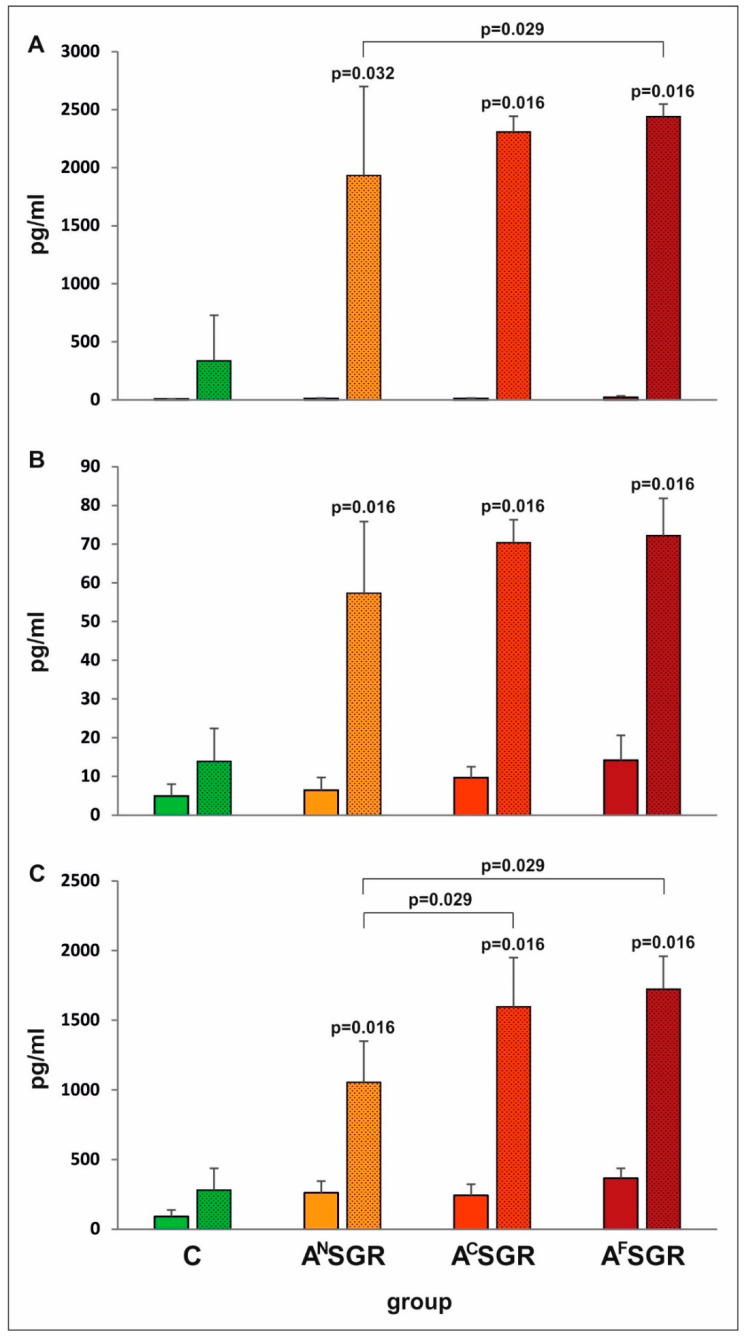
In vitro IFN-γ (**A**), IL−2 (**B**) and IL−10 (**C**) cytokine release by splenocytes from immunized and control mice in unstimulated (plain bars) or TLA-stimulated (dotted bars) cultures. *p* values are provided above the bars to indicate significant differences in cytokine synthesis between TLA-stimulated splenocytes from immunized and control nonimmunized mice (C); statistically significant differences between the TLA-stimulated cultures of immunized groups are marked with brackets.

The mouse immunization with any of the three tetravalent chimeric antigens led to the induction of a strong specific immune response characterized by the in vitro release of Th1 (IFN-γ and IL−2) and Th2 (IL−10) cytokines in response to antigenic stimulation (TLA). Regardless of the cytokine being evaluated, the concentrations measured in the supernatants of stimulated splenocytes from vaccinated mice were significantly higher than those measured in the supernatants of corresponding cultures of control splenocytes from nonimmunized mice (Figure 3). Furthermore, the release of cytokines by splenocytes from immunized mice in response to TLA stimulation was statistically higher than that by splenocytes in the presence of medium alone for all tested experimental groups (*p* = 0029 regardless of the cytokine or immunized group under study).

The analysis of cytokine production revealed certain differences among the experimental groups. Splenocytes from all vaccinated mice, regardless of the portion of AMA1 present in the chimeric protein structure, released comparable amounts of IL−2 after antigenic stimulation (*p* > 0.400). On the other hand, immunization with A^N^SGR resulted in less pronounced production of IL−10 and IFN-γ by TLA-stimulated splenocytes in vitro compared to either of the two other chimeric antigen immunizations (*p* = 0.029) or A^F^SGR immunization only (*p* = 0.029), respectively.

## 4. Discussion

As stated above, despite many years of research, toxoplasmosis still poses a significant risk to immunocompromised individuals and causes considerable economic losses in animal husbandry. Taking into account the shortcomings of currently used drugs that were not specifically designed to treat *T. gondii* infection, cause substantial side effects and are essentially ineffective against the dormant parasite stage enclosed within tissue cysts, the development of an efficient anti-*Toxoplasma* vaccine is of paramount importance. The most desirable outcome would be the construction of a universal vaccine capable of protecting all possible hosts from both acquired and congenital toxoplasmosis. In other words, the vaccine should reduce or optimally block parasite transmission to all possible hosts, including humans, by preventing the infection of meat-producing animals and oocyst-shedding felids, the definitive hosts of the parasite. Since this goal remains elusive, the aim of the present study was to test the immunoprophylactic utility of three tetravalent chimeric *T. gondii* proteins, as the use of recombinant proteins for vaccine development represents one of the most common, modern and safe approaches for immunoprophylaxis used in several commercially available vaccines for humans [44,45].

As noted in our previous work on chimeric antigens as potential vaccine candidates, according to many studies, multiantigenic compositions seem to be much more effective in generating protective immunity in vaccinated individuals than single proteins. Moreover, chimeric antigens constructed as recombinant proteins constitute a well-defined, fairly inexpensive and relatively safe tool for vaccine development due to their high purity [35].

The current work represents a continuation of previously carried out experiments that led to the selection of a trivalent chimeric antigen based on the SAG2, GRA1 and ROP1 *T. gondii* antigens (SGR) as a promising vaccine candidate for further research on toxoplasmosis immunoprophylaxis. The trivalent antigen was capable of triggering strong protective immunity in vaccinated and parasite-challenged mice, which was defined by an 86% decrease in the brain cyst load [35]. Although the elucidated protection was high, it was not complete; thus, the next step was to further boost the antigen’s immunoprotective capacity by incorporating another antigen into the chimeric structure. The experimental design was based on the observation that recombinant proteins used concomitantly often demonstrate a synergistic effect that triggers a protective response in vaccinated animals [46]. Furthermore, the AMA1 antigen, assigned to the microneme proteins, is considered to play a critical role in host cell invasion. In general, microneme proteins, which are released upon contact of the parasite with the host cell membrane, are involved in several processes including recognition of and adhesion to the host cell and the subsequent disturbance of host cell integrity, which are indispensable for host cell invasion and thus *T. gondii* replication and survival. The AMA1 antigen is believed to interact with rhoptry neck proteins (RONs)—RON2, RON4, RON5, and RON8—to form the MJ complex, which is believed to be pivotal for adhesion and host cell penetration [36,37,38]. Also, the use of this protein for immunization, delivered as a peptide or DNA vaccine, has been shown to promote survival and limit cyst formation in *T. gondii*-infected mice [39,40,41]. Interestingly, the *T. gondii* RH-AMA1 knockout parasites seem avirulent for immunocompetent mice, which underlines the importance of the protein for the expansion of the parasite in vivo [47]. Finally, the AMA1 protein of another Apicomplexan parasite *P. falciparum* has been proposed as a promising vaccine component and AMA1 antigen-based formulations even advanced to Phase 2 clinical trials in malaria endemic areas in Africa [48,49].

The results of the present study clearly show that all three tested chimeric antigens that contained different portions of the AMA1 antigen were immunogenic, as their administration led to the development of strong humoral and cellular responses in vaccinated mice. All immunized mice produced specific IgG antibodies that reacted strongly with the components of *Toxoplasma* lysate antigen, indicating that immunoglobulins induced by recombinant proteins were capable of recognizing the corresponding native equivalents. Further analysis of synthetized IgG antibody subclasses revealed the production of both IgG1 and IgG2a antibodies, which proves the induction of Th2 and Th1 immune responses, respectively, and there was a predominance of the IgG1 isotype as noted previously [35]. Interestingly, the AMA1 antigen delivered as a DNA vaccine has been reported to preferentially trigger IgG2a synthesis in immunized mice compared to IgG1 [39].

Despite the generally comparable humoral responses, which were defined by specific IgG antibodies, in mice following vaccination, the evaluation of the proliferative response and cytokine release revealed pronounced differences in the immunogenic activities of the tested chimeric proteins. Only splenocytes from mice immunized with the A^C^SGR or A^F^SGR chimeric protein, which contained domains II and III and the full-length AMA1 antigen, respectively, exhibited a proliferative response in vitro upon TLA stimulation that was stronger than the response measured for splenocytes from nonvaccinated controls. Additionally, splenocytes from these two experimental groups cultured in the presence of native *T. gondii* antigens released more cytokines, typically assessed in anti-*T. gondii* vaccine studies and considered indicative of Th1 (IL−2 and IFN-γ) and Th2 (IL−10) immune responses than those from the group vaccinated with A^N^SGR, which contained only domain I of AMA1, although not all noticed differences were statistically significant.

Similar observations were made in an in vivo protection assay in which the A^N^SGR antigen provided the weakest protection against chronic toxoplasmosis in mice (38%). Immunization with the A^C^SGR or A^F^SGR chimeric protein triggered immunoprotective immunity, which led to decreases in the brain cyst load of 61% and 67%, respectively. Intriguingly, these observations overlap with the results of the assessment of the diagnostic value of both AMA1 fragments and chimeric antigens containing different AMA1 regions [42,50]. It needs to be emphasized that the employment of recombinant proteins in vaccine construction is based on the assumption that specific components of the immune system produced in response to stimulation with a recombinant protein are able to recognize and react with the native equivalent present in the invading infectious agent. Thus, all chimeric antigens used in our experiments were tested concomitantly as potential vaccines and diagnostic tools. This approach was very productive since it led to the selection of the SGR protein as a very promising vaccine candidate. The possible explanations for the lower reactivity of the N-terminal fragment of AMA1 compared to the C-terminal portion and the full-length protein, which was discussed extensively in regard to the diagnostic utility of this protein, may in fact also refer to the immunoprophylactic capacity of tested AMA1-containing chimeric antigens. Thus, the varied performances of the tested recombinant proteins might be explained by the engagement of the specified portions of AMA1 in the invasion process and their availability to the immune system. The N-terminal fragment of AMA1 corresponding to domain I of the antigen contains residues involved in formation of a binding pocket for the loop region of RON2 protein during the formation of the MJ complex [51,52]. Thus, it might be speculated that the contact of this portion of AMA1 with components of the immune system is limited, on the other hand, the C-terminal fragment corresponding to domains II and III might be more accessible [42,50,53,54]. Another possibility is the lower immunogenicity of AMA1 domain I, however, this portion of the antigen contains at least one predicted T cells epitope (amino acid residues 197–204) [40]. Since there are few hypotheses involving the AMA1 protein and its exact performance in the mechanism underlying *T. gondii* invasion, this protein remains an interesting object of studies at both the theoretical and application levels.

Nevertheless, out of all tested antigenic variants containing different fragments of AMA1, the chimeric protein incorporating the full-length AMA1 antigen was the most efficient in terms of both triggering potent humoral and cellular immune responses and providing protection against chronic toxoplasmosis in mice. This observation supports our previous observation that longer fragments of recombinant proteins in a chimeric structure are more immunogenic and exhibit a higher immunoprotective capacity than shorter fragments [35]. Noteworthy, it has been shown that full length AMA1 can trigger strong immunity in mice resulting in significantly enhanced survival and partial protection against cyst formation [39,41]. Disappointingly, although our goal was to boost the immunoprotective potential of the SGR protein by the addition of the AMA1 antigen, the resulting protein actually demonstrated lower activity than its trivalent counterpart; this phenomenon was observed previously in regard to the addition of GRA2 to the SGR core [35]. It has even been shown that two plasmids capable of inducing protective immunity when administered separately completely cancel the immunoprotective effect when injected concomitantly [55]. It is also important to remember that although chimeric proteins possess several unquestionable advantages over separate recombinant antigens, they also represent somewhat artificial constructs with unpredictable immunogenicity [56], and thus, each single preparation must be thoroughly evaluated in vitro and in vivo to draw any compelling conclusions.

## 5. Conclusions

The results of present work underline again the impact of the antigenic composition of recombinant chimeric proteins, mostly in regard to the length of incorporated fragments, on their immunogenic and immunoprotective activities. Furthermore, although the eradication of *T. gondii* relies primarily on the cellular response with antibodies providing support, the approach to immunoprophylaxis based on evaluating the humoral response to determine the diagnostic utility of vaccine candidates proposed in our studies seems highly productive and efficient. Indeed, regardless of the predominant immunity type involved in protection, both cellular and humoral mechanisms are indispensable, complement one another and work synergistically during parasite invasion.

## Figures and Tables

**Table 1:** Titers of IgG1 and IgG2a antibodies in the serum of vaccinated mice.

Mouse/Group	Antibody Titer		
	IgG1	IgG2a	IgG1/IgG2a
**A^N^SGR**			
mouse 1	>204,800	51,200	>4/1
mouse 2	204,800	25,600	8/1
mouse 3	204,800	6400	32/1
mouse 4	102,400	6400	16/1
**A^C^SGR**			
mouse 1	>204,800	12,800	>16/1
mouse 2	204,800	6400	32/1
mouse 3	>204,800	6400	>32/1
mouse 4	204,800	6400	32/1
**A^F^SGR**			
mouse 1	>204,800	25,600	>8/1
mouse 2	>204,800	102,400	>2/1
mouse 3	204,800	25,600	8/1
mouse 4	204,800	12,800	16/1

**Table 2:** Proliferative responses of splenocytes from vaccinated and control mice stimulated with TLA in vitro.

Antigen/Group	Mean OD^570^ ± SD ^a^			
	Control	A^N^SGR	A^C^SGR	A^F^SGR
Medium	0.254 ± 0.048	0.249 ± 0.021	0.215 ± 0.043	0.263 ± 0.058
TLA	0.505 ± 0.048	0.505 ± 0.038	0.542 ± 0.039	0.550 ± 0.044 ^a^

^a^ statistically significant differences compared to control (PBS/IFA–injected mice).
